# Melena as Initial Presentation of Adenocarcinoma in Pancreatic Tail

**DOI:** 10.7759/cureus.1744

**Published:** 2017-10-04

**Authors:** Stella Pak, Damian Valencia, James Kim, Christine Dee

**Affiliations:** 1 Internal Medicine, Kettering Medical Center; 2 Department of Medicine, Kettering Medical Center; 3 Wright State University Boonshoft School of Medicine

**Keywords:** melena, pancreatic cancer, splenic vein thrombosis, adenocarcinoma

## Abstract

Melena associated with gastric varices, in the setting of possible obstructing pancreatic adenocarcinoma, has been poorly documented as an initial presenting sign for pancreatic malignancy. Considering the late presentation of patients with pancreatic malignancy, it is important to consider all potential presenting symptoms for the early detection and treatment of pancreatic malignancy. Below, we present a patient with no history of liver pathology, who presents with melena and gastric varices, likely relating to portal hypertension in the setting of an obstructing pancreatic malignancy.

## Introduction

Cancer is one of the leading causes of mortality in the United States. Pancreatic cancer is the fourth leading cause of death due to its limited treatment options and delayed diagnosis. The annual incidence of pancreatic cancer in the United States is about 7.4 per 100,000 people. The vast majority (95.5%) of pancreatic tumors are nonfunctional and present with symptoms primarily caused by mass effect [1]. Typical clinical symptoms include jaundice, epigastric pain, vague abdominal symptoms, back pain, anorexia, nausea, vomiting, weight loss, dark urine, and light stool [2]. Due to these nonspecific and late-onset symptoms, pancreatic cancer is often diagnosed in the advanced stage. Pancreatic cancers, in general, have a very poor prognosis, with the overall five-year survival rate being about 4% [2]. Below, we present a case where pancreatic tail malignancy initially presents with melena. 

## Case presentation

A 69-year-old woman with chronic melena and diverticulosis presented with two weeks' duration of worsening shortness of breath on exertion and fatigue. She had a blood pressure of 108/65 mmHg, heart rate of 89/min, respiration rate of 12/min, body temperature of 98.3 °F, and oxygen saturation of 100% on room air. Physical examination was notable for pale appearance and dry mucous membranes and skin. Her abdomen was soft without hepatosplenomegaly. Laboratory investigation was remarkable for hemoglobin of 6.4 g/dL and a positive fecal occult blood test. She was expeditiously transfused with two units of blood and hydrated vigorously with intravenous fluid.

**Figure 1:**
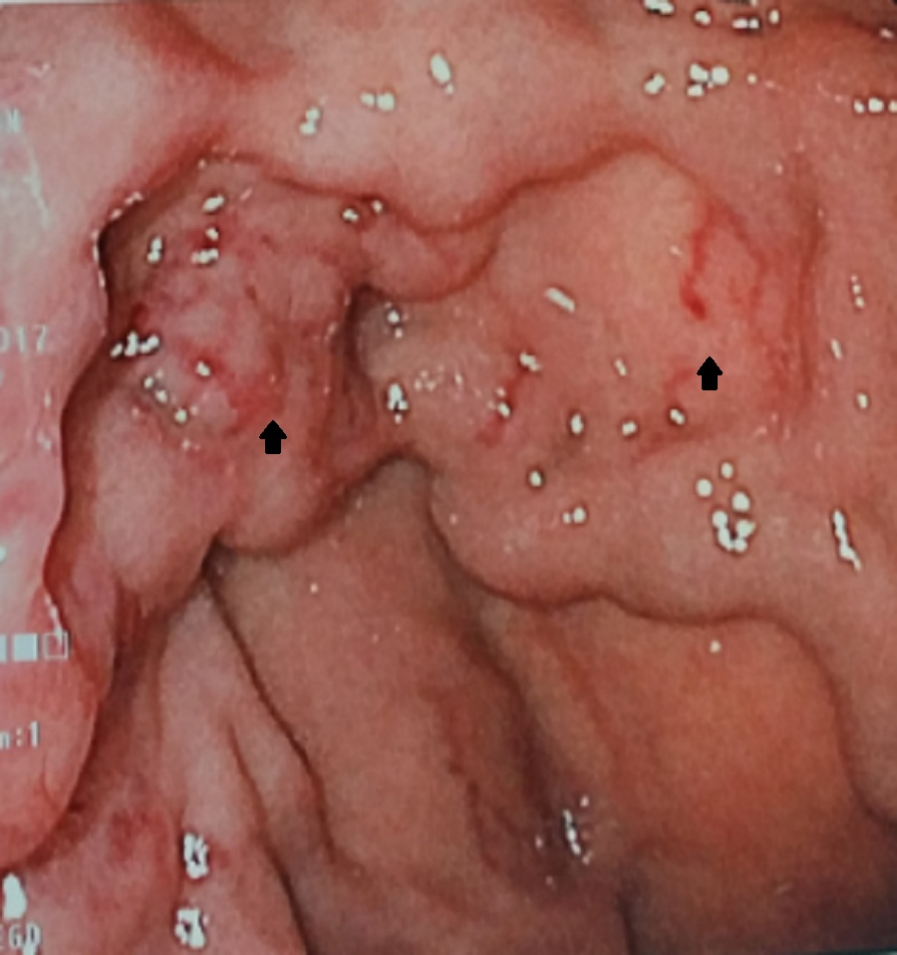
Upper endoscopy showing prominent gastric varices in the gastric cardia

**Figure 2:**
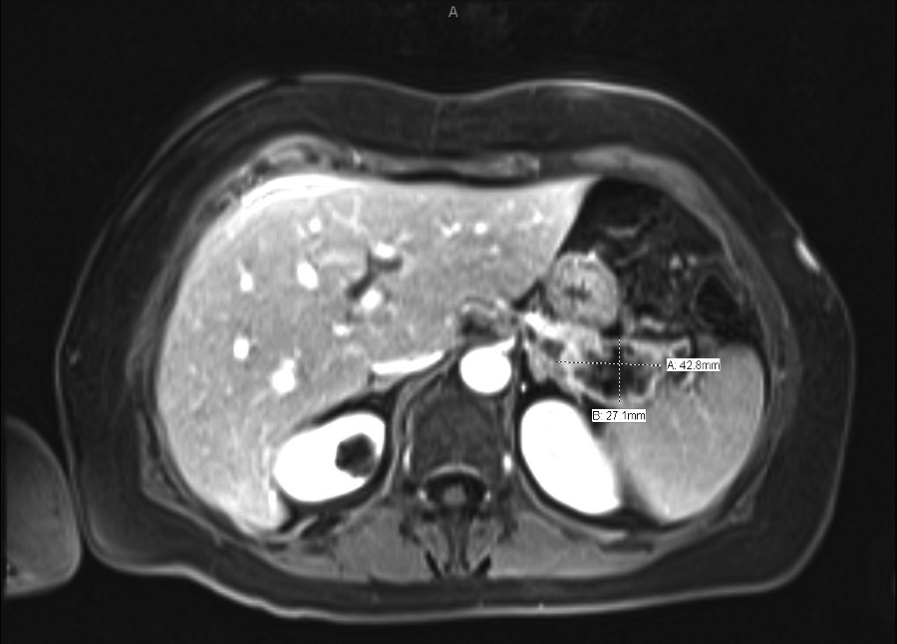
Magnetic resonance imaging demonstrating adenocarcinoma (27.1 mm X 42.8 mm) in the tail of the pancreas

Colonoscopy showed diverticulosis, which was consistent with her known medical history. Upper endoscopy showed prominent varices in the gastric cardia (Figure 1). A computerized tomography (CT) scan of the abdomen and pelvis showed acute sigmoid diverticulitis and a hypoenhancing region in the tail of the pancreas. This incidental finding on the CT scan prompted a further assessment of the region with magnetic resonance imaging (MRI). The MRI revealed a thin-walled, peripherally enhancing lesion with enhancing internal septa and adjacent stranding (Figure 2). Additionally, splenic vein thrombosis and multiple portosystemic collaterals were found on MRI. Endoscopic ultrasound-guided fine needle aspiration yielded malignant cell clusters that resemble adenocarcinoma. In accordance with the patient’s wish, palliative care was pursued with symptomatic management.

## Discussion

Considering this patient's history of diverticulosis, it was difficult to assess whether or not melena was related to pancreatic malignancy. This patient's positive findings included anemia with several associated symptoms, including progressive shortness of breath, pale skin, and dry mucous membranes are all associated with the reported history of melena. Hematochezia and melena have been previously documented as rare presenting signs of pancreatic malignancy, both symptoms as a result of malignant invasion and erosion into the gastrointestinal lumen. In the patient case presented above, endoscopic gastric duodenoscopy revealed several varices in the gastric cardia, which can be a result of pancreatic malignancy causing obstructive portal hypertension. Considering this patient has had no history of liver pathology or other cause of portal vein obstruction, it is likely that these gastric varices are a direct result of obstructing pancreatic malignancy. Although the patient presented with melena and gastric varices, which are uncommon signs of pancreatic malignancy, it is important to consider further evaluation, specifically in patients without a history of liver pathology.

The phenomenon described in this case report, causing the patient’s ruptured gastric varices is known as pancreatic sinistral portal hypertension (PSPH). First described by Greenwald and Wasch in 1939, pancreatic sinistral portal hypertension is a rare syndrome that occurs with an incidence of <1% [3] and accounts for <5% of patients with portal hypertension. Sinistral portal hypertension, by definition, is a type of portal hypertension that occurs on the left-sided segment of the portal venous system (sinistral: left-sided or segmental). Even though PSPH occurs in the splenic vein, the portal pressure is, in fact, within the normal range, therefore, making the term “sinistral portal hypertension” a misnomer [4].

The most likely cause of PSPH is splenic vein thrombosis, which was seen in this patient. Other causes of PSPH, although also extremely rare, include complications of liver transplantation, partial gastrectomy, retroperitoneal fibrosis and tuberculosis, perirenal abscess, and hereditary thrombophilias. Other associations of splenic vein thrombosis include chronic pancreatitis, pancreatic pseudocyst, hypercoagulable state trauma, peptic ulcer disease, retroperitoneal fibrosis, and pancreatic tuberculosis [3,5-6]. In this scenario, thrombosis tends to occur largely due to the proximity of the splenic vein with the pancreas. Whether there is cancer or inflammation, compressions or spasms of the splenic vein may then cause stasis of venous blood, leading to intimal damage and then thrombus formation [3]. If a pancreatic tumor is present, it tends to be on the tail. In splenic vein thrombosis with PSPH, there tends to be a triad on presentation as well, which includes isolated gastric varices, splenomegaly, and normal liver function [3]. However, these features are not always strictly present together. The patient, in this case, did not present with splenomegaly. It was initially believed that splenomegaly was a universal feature of PSPH. However, it was found to have been absent in multiple reports. In addition, the literature states that 45–72% present with upper gastrointestinal bleeds secondary to varices and 25–38% complain of abdominal pain. However, in another study, approximately 15% of patients with splenic vein thrombosis presented with variceal bleeding [7].

Therapeutically, patients with upper gastrointestinal (GI) bleed along with sinistral or left-sided portal hypertension can be managed by means of surgical intervention. Any correction is geared towards the underlying cause, whether it may be a pancreatic neoplasm, for example, and splenectomy would also be a general part of surgical management. Essentially, the goal is to decrease the arterial into the left portal system. However, the degree of left-sided portal hypertension makes the dissection of the branches of the splenic vessels difficult and risky during a laparoscopic splenectomy. Recently, splenic arterial embolization preoperatively to laparoscopic splenectomy has become a safer and simpler practice by decreasing the portal pressure [8]. As for the upper GI bleed itself, endoscopic therapy can be done and, in asymptomatic patients, there is much controversy pertaining to surgical intervention. However, conservative management is likely to be considered as an acceptable option.

Nevertheless, PSPH, as described in this case report, encourages raising concern about pancreatic cancers in those with gastric varices. In general, considering that varices led to the discovery of an underlying pancreatic cancer in this case, it is also important to note that with a known diagnosis of pancreatic cancer, varices can be a complication [3]. Having such knowledge can promote awareness of this rare presentation of pancreatic cancer, thereby allowing early diagnosis and intervention.

Regarding the nature of pancreatic cancer itself, the lifetime risk is approximately 1.3%. The sensitivity of tumor markers, such as carcinoembryonic antigen (CEA) and carbohydrate antigen 19-9 (CA 19-9), are not considered reliable for screening. Other general workup includes dual-phase, contrast-enhanced spiral CT, which is the imaging modality of choice. Endoscopic retrograde cholangiopancreatography (ERCP) or magnetic resonance cholangiopancreatograph (MRCP) may be useful for assessing any biliary or pancreatic duct dilation. A biopsy can also be done, although this is not always necessary if imaging is highly suggestive of malignancy. If localized, surgery is done, which includes doing a modified Whipple procedure if the tumor is on the pancreatic head or doing a pancreatectomy (which normally includes splenectomy) if the tumor is present on the body or tail. In metastatic disease, gemcitabine historically has been the chemotherapy agent of choice [9]. Options for screening remain limited. However, according to recent literature, attempts have been made to find possible early detection biomarkers for pancreatic ductal adenocarcinoma. Circulating long non-coding ribonucleic acids (RNAs) (lncRNAs) is one that has recently been investigated, which has demonstrated some potential to detect precursors to pancreatic ductal adenocarcinoma, such as intraductal mucinous papillary neoplasm (IPMNs) [10].

## Conclusions

It is difficult to know if this patients' melena was a direct consequence of her history of diverticulosis or a manifestation of early progressive pancreatic adenocarcinoma. In any case, it is important to consider melena with findings of gastric varices in a patient without a history of liver pathology as a potential, initial presenting symptom for early detection and treatment of pancreatic malignancy.
